# Efficacy of Acupuncture Treatment for Postprandial Distress Syndrome: A Systematic Review and Meta-Analysis

**DOI:** 10.1155/2022/6969960

**Published:** 2022-06-02

**Authors:** Jihang Du, Yinhao Feng, Qiang Yuan, Haiping Gong, Jie An, Liu Wu, Qian Dai, Bojun Xu, Haozhong Wang, Jian Luo

**Affiliations:** ^1^Hospital of Chengdu University of Traditional Chinese Medicine, Chengdu 610072 Sichuan, China; ^2^School of Acupuncture and Tuina, Chengdu University of Traditional Chinese Medicine, Chengdu 610075, China; ^3^Departments of Acupuncture and Massage, Changchun University of Chinese Medicine, Changchun 130117 Jilin, China; ^4^College of Basic Medicine, Chengdu University of Traditional Chinese Medicine, Chengdu 610075, China

## Abstract

**Objective:**

This systematic review and meta-analysis was conducted to assess the efficacy of acupuncture treatment for postprandial distress syndrome (PDS).

**Methods:**

Search the Web of Science, the Cochrane Library, PubMed, and Embase databases with acupuncture randomized controlled trials for the treatment of patients with PDS. Strictly according to inclusion and exclusion quality assessment standards, the qualified ones are used to study the optimum extraction and data by two independent reviewers. Stata 15.0 software was used for meta-analysis.

**Result:**

We initially identified 63 studies, of which five (1253 participants) were eventually included in our analysis. There were 643 cases in the experimental group and 610 cases in the control group. Acupuncture had a significant effect on the total therapeutic effect (OTE) at week 4 (OR 4.74, 95% CI 02.88-7.83, *Z* = 6.10, *P* = 0 < 0.05). Significantly improved NDI (Nepean dyspepsia index) scores of PDS patients at week 4 (SMD 0.61, 95% CI 0.48 to 0.74). Significantly improved NDI scores in PDS patients at week 16 (SMD 0.49, 95% CI 0.27 to 0.71). After acupuncture treatment, the SID (dyspepsia symptom index) score of PDS patients decreased significantly at week 4 (SMD-0.52, 95% CI -0.73 to -0.32) and week 16 (SMD-0.59, 95% CI -0.81 to -0.36). Postprandial satiety scores (SMD-0.63, 95% CI -0.76 to -0.50) and early satiety scores (SMD-0.51, 95% CI -0.64 to -0.37) were also significantly lower at week 4 after acupuncture.

**Conclusion:**

This study highlighted that the acupuncture could significantly improve the overall therapeutic effect of PDS patients, alleviate the symptoms of postprandial fullness and early satiety, and improve the quality of life of patients. Our results supported that acupuncture was an effective therapeutic strategy for postprandial distress syndrome.

## 1. Introduction

Functional dyspepsia (FD) often presents with predominant pain in association with symptoms of early satiation and postprandial fullness [1]. The prevalence of functional dyspepsia is approximately 16%, but according to different regions and diagnostic criteria, it might be different [2]. It has a serious impact on the quality of life and social functions [3, 4]. According to a survey, among participants with Rome IV FD, postprandial distress syndrome (PDS) was the most common subtype, accounting for 61% of all functional dyspepsia, with epigastric pain syndrome accounting for 18% and the overlapping variant accounting for 21% [5]. In the United States in 2009, a conservative estimate of the cost of FD treatment was $18.4 billion [6], causing a huge economic burden on public health. Treatment approaches include eradication of helicobacter pylori, acid suppression therapy, prokinetic drugs, and central neuromodulators [2]. As a part of Traditional Chinese medicine, acupuncture has been used as an alternative therapy to treat various diseases, including gastrointestinal diseases. Acupuncture can directly regulate gastrointestinal motility, regulate the secretion of brain gut peptide, activate n-methyl-D-aspartic acid (NMDA) receptor, and regulate signaling pathways, which has been widely used in gastrointestinal diseases. A randomized controlled trial has shown that acupuncture is an effective nondrug treatment for FD [7]. Meanwhile, a retrospective review showed that patients with PDS responded better to acupuncture therapies, and acupuncture could effectively relieve postprandial fullness and early satiety symptoms of PDS patients [8]. Acupuncture can regulate the enteral intermuscular nerve plexus, enteral gangliocytes, Cajal interstitial cells (ICC), and neurotransmitters and their receptors. Electroacupuncture can improve dyspepsia symptoms and gastrointestinal motility disorders by regulating the vagal nerve and gastrointestinal hormone mechanism. Electroacupuncture “Zusanli point” can reduce the gastric residual rate of functional dyspepsia, increase the protein content and MR-NA expression of SCF and C-kit in gastric antrum, and improve the structure and morphology of ICC and intestinal smooth muscle cell (SMC). These results indicate that acupuncture can regulate gastrointestinal motility by regulating the SCF/C-KIT signaling pathway. Several clinical trials have assessed the efficacy of acupuncture for PDS and have not been satisfactory. It is considered that there are controversies about the clinical efficacy of acupuncture in the treatment of PDS. We conducted this systematic review and meta-analysis using existing randomized controlled trials to determine the effects of acupuncture on SID, NDI, and OTE in patients with PDS.

## 2. Methods

### 2.1. Search Strategy and Selection Criteria

This study was reported according to the Preferred Reporting Program for Systematic Evaluation and Meta-Analysis (PRISMA) statement and was registered in the International Registry for Prospective Systems Evaluation (CRD42022292889) on January 11, 2022. We selected relevant studies by searching Cochrane, Embase, Web of Science, and PubMed. We used the following combined text and MeSH terms: “acupuncture” and “postprandial distress syndrome.” The complete search used for PubMed was as follows: ((“Acupuncture”[Mesh]) OR ((acupuncture [Title/Abstract]) OR (Pharmacopuncture [Title/Abstract]))) AND (Postprandial distress syndrome [Title/Abstract]). We considered all potentially eligible studies for review. In addition a manual search was performed by using the reference lists of key articles published in English.

### 2.2. Study Selection and Data Extraction

RCT in which all the participants met the diagnostic criteria for PDS as recommended by Rome IV or Rome III with normal gastroscopy results. The selected subjects did not have serious or malignant diseases that caused dyspepsia symptoms and did not take drugs that might affect dyspepsia. The experimental group was treated with acupuncture, while the control group was treated with sham acupuncture (fake acupuncture point acupuncture contrast method, the symptoms related to acupuncture point comparison method, and false hole shallow thrust method). Exclusion criteria were as follows: (1) The patient suffered from other serious or malignant diseases that might cause dyspepsia, such as liver cirrhosis, heart failure, or gastrointestinal tumors; (2) taking drugs that might affect dyspepsia in the past 1 month, such as acid suppressors, stimulants, nonsteroidal anti-inflammatory drugs, and antidepressants; (3) full text or original research data were not available; (4) case reports, abstracts, conference papers, and other nonpublished literatures, reviews, and meta analyses; and (5) repeated publication.

The outcomes assessed were as follows: randomized controlled trials or retrospective studies which reported change in SID or NDI between baseline and the end of intervention, proportion of patients with significant or moderate remission of symptoms at the end of intervention, and changes in postprandial satiety and early satiety symptoms between baselines and the end of the intervention.

2 independent investigators (HJD, PHG) reviewed titles and abstracts, studies which met the inclusion criteria were selected for full-text assessment. Trials selected for detailed analysis and data extraction was carried out independently by 2 investigators (HJD and QY) with an agreement value (*κ*) of 96·5%. Disagreements between reviewers on inclusion were resolved by a third investigator (QD).

We extracted the following data from each selected study: age, total number of participants, disease duration, change in NDI and SID (mean (SD)), OTE (OR), change in postprandial fullness, and early satiety at end of intervention (mean (SD)). Assessments of risk of bias were completed by two independent reviewers according to PRISMA recommendations.

### 2.3. Statistical Analysis

Stata (version 15.0) were used for all statistical analyses. We assessed the effect of acupuncture treatment according to the five outcomes: change in NDI and SID (mean (SD)), change in postprandial fullness and early satiety (mean (SD)), and OTE (OR). NDI, SID, postprandial fullness, and early satiety were continuous variables, and OTE was dichotomous variables.

Dichotomous data were expressed as relative risk (OR) and continuous variables as standardized mean difference (SMD) with 95% confidence interval (CI). The *χ*^2^ test and *I*^2^ statistics were used for the assessment of heterogeneity. A fixed-effects model was used if there was no obvious heterogeneity (*I*^2^ ≤ 50% or *P* > 0.05). If the heterogeneity was significant, the source of heterogeneity was identified by conducting subgroup analysis or sensitivity analysis. Publication bias was not performed because the number of the studies in a group was less than 10.

## 3. Results

### 3.1. Literature Search and the Characteristics of the Studies

**Figure 1:**
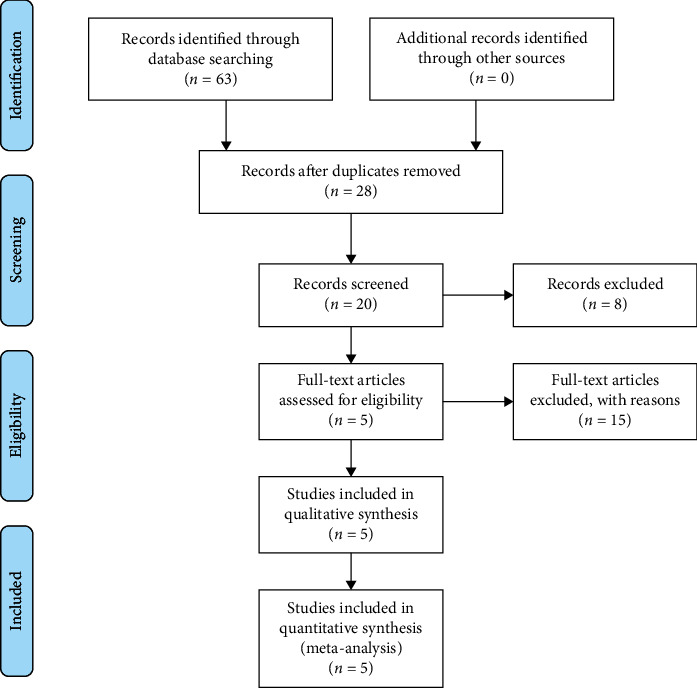
Flow chart.

We identified 63 studies, of which 5 were included in our analysis (Figure 1). The trials were all published between 2015 and 2021 (one was published in 2015, one was published in 2019, two were published in 2020, and one was published in 2021) (Table 1) [8–12]. All the trials were conducted in China. Three articles [8, 10, 11] were published in English and 2 [9, 12] in Chinese. All RCTs adopted a parallel-group design, among which four studies employed two parallel-arm group designs [9–12], and one used six parallel-arm group designs [8]. In all trials, acupuncture or electroacupuncture was used in the experimental group and sham acupuncture was used in the control group (Table 1).

### 3.2. Risk of Bias

**Figure 2:**
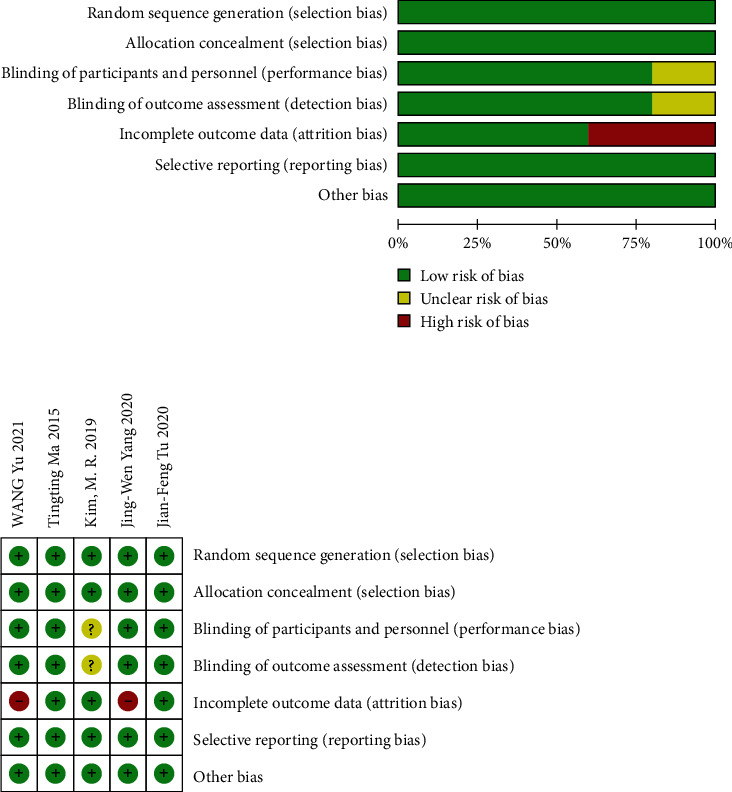
Risk of bias.

All the literatures included in this meta-analysis were randomized controlled trials. The practitioners of acupuncture were not blinded to the trial, because there was obvious operational difference between acupuncture and sham acupuncture. This did not significantly affect the research results; thus, it was considered to be low risk. One study [9] did not describe blindness in detail; thus, it was evaluated without unknown risk bias, and two [11, 12] studies had a high loss of follow-up bias (Figure 2).

### 3.3. Meta-Analysis Results

#### 3.3.1. Outcome A: Change of NDI from Baseline to Week 4

**Figure 3:**
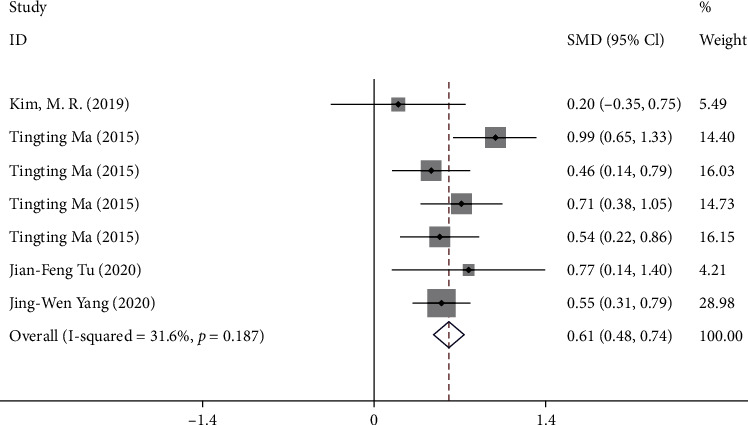
Forest plot of comparison. Acupuncture vs. sham acupuncture. Outcome: change of NDI from baseline to week 4.

Four trials [8–11] reported the change in NDI from baseline to week 4. One trial [9] reported the NDI at week 4. A total of 975 patients were included in the analysis (*P* = 0.19, *I*^2^ = 31.6% < 50%). The pooled results showed that compared with sham acupuncture, acupuncture could significantly improve NDI score in patients with postprandial distress syndrome (SMD 0.61, 95% CI 0.48 to 0.74) (Figure 3). No significant publication bias was found by the funnel plot and Begg test (*P* = 0.96 > 0.05).

#### 3.3.2. Outcome B: Change in NDI from Baseline to Week 16

**Figure 4:**
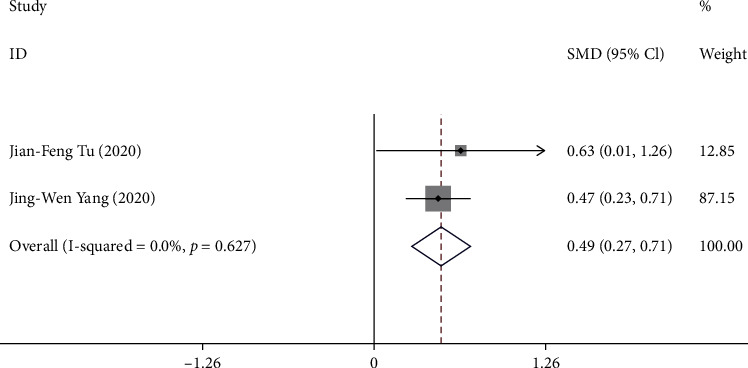
Forest plot of comparison. Acupuncture vs. sham acupuncture. Outcome: change in NDI from baseline to week 16.

Two trials [10, 11] reported the change in NDI from baseline to week 16. A total of 320 patients were included in the analysis. The pooled results showed that compared with sham acupuncture, acupuncture can significantly improve the NDI score of patients with postprandial distress syndrome at week16 (SMD 0.49, 95% CI 0.27 to 0.71) with low heterogeneity (*I*^2^ = 0.0%, *P* = 0.63) (Figure 4).

#### 3.3.3. Outcome C: Change in SID from Baseline to Week 4

**Figure 5:**
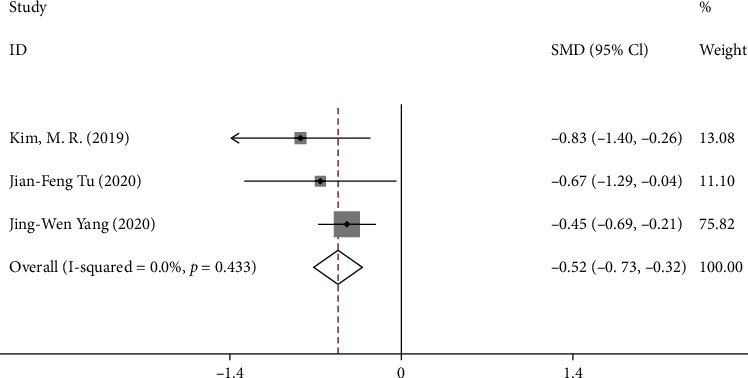
Forest plot of comparison. Acupuncture vs. sham acupuncture. Outcome: change in SID from baseline to week 4.

Three trials [9–11] reported the change in SID from baseline to week 4. A total of 371 patients were included in the analysis. The pooled results showed that compared with sham acupuncture, acupuncture could significantly reduce the SID score of the patients with postprandial distress syndrome at week 4 (SMD -0.52, 95% CI -0.73 to -0.32), with low heterogeneity (*I*^2^ = 0.0%, *P* = 0.43) (Figure 5).

#### 3.3.4. Outcome D: Change in SID from Baseline to Week 16

**Figure 6:**
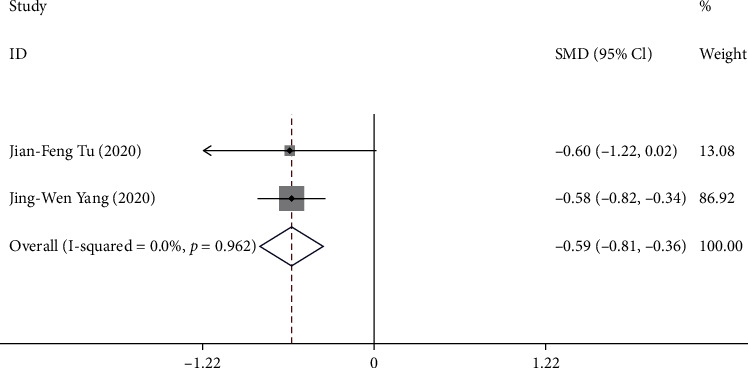
Forest plot of comparison. Acupuncture vs. sham acupuncture. Outcome: change in SID from baseline to week 16.

Two trials [10, 11] reported the change in SID from baseline to week 16. A total of 320 patients were included in the analysis. The pooled results showed that compared with sham acupuncture, acupuncture could significantly reduce the SID score of patients with postprandial distress syndrome at week 16 (SMD -0.59, 95% CI -0.81 to -0.36) with a low heterogeneity (*I*^2^ = 0.0%, *P* = 0.96) (Figure 6).

#### 3.3.5. Outcome E: OTE at Week 4

**Figure 7:**
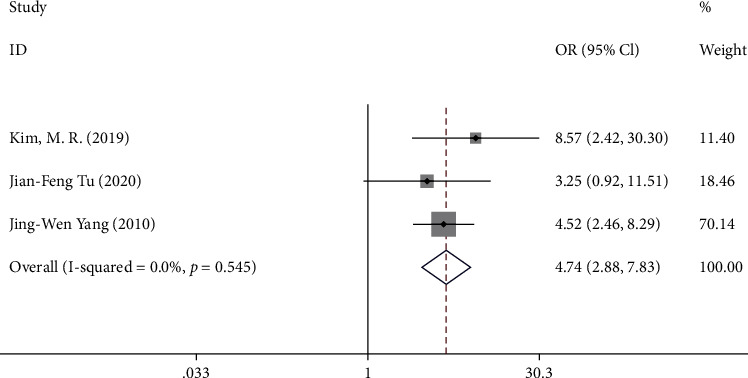
Forest plot of comparison. Acupuncture vs. sham acupuncture. Outcome: OTE at week 4.

Three trials [9–11] reported OTE at week 4. A total of 371 patients were included in the analysis. The pooled results showed that compared with sham acupuncture, acupuncture could significantly enhance the overall treatment efficacy (OTE) at week 4 (OR 4.74, 95% CI 2.88 to 7.83, *Z* = 6.10, *P* = 0 < 0.05) with a low heterogeneity (*I*^2^ = 0.0%, *P* = 0.55) (Figure 7).

#### 3.3.6. Outcome F: Change in Postprandial Fullness Score from Baseline to Week 4

**Figure 8:**
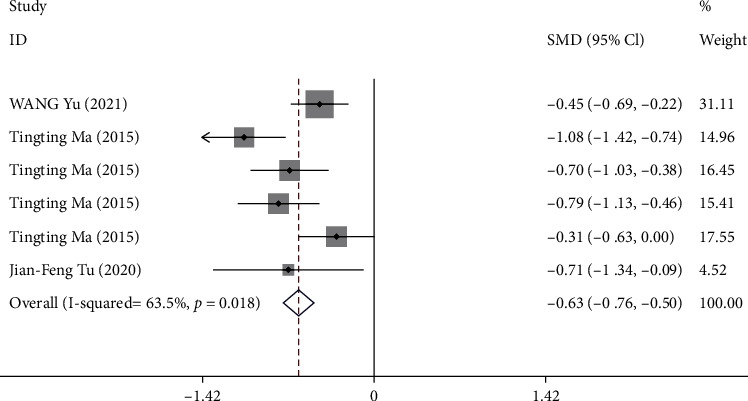
Forest plot of comparison. Acupuncture vs. sham acupuncture. Outcome: change in postprandial fullness score from baseline to week 4.

Three trials [8, 10, 12] reported the change in postprandial fullness score from baseline to week 4. One trial [12] reported the postprandial fullness score at week 4. A total of 768 patients were included in the analysis with high heterogeneity (*P* < 0.05, *I*^2^ = 63.5%). The pooled results showed that compared with sham acupuncture, acupuncture could significantly reduce the postprandial fullness score at week 4 (SMD-0.63, 95% CI -0.76 to -0.50) (Figure 8).

#### 3.3.7. Outcome G: Change in Early Satiety from Baseline Score to Week 4

**Figure 9:**
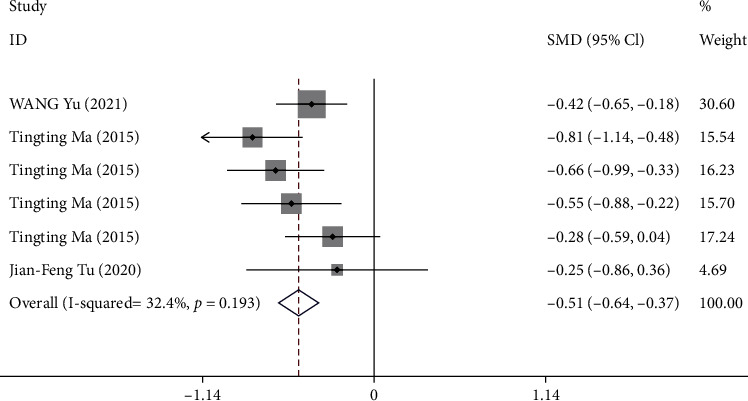
Forest plot of comparison. Acupuncture vs. sham acupuncture. Outcome: change in early satiety from baseline score to week 4.

Three trials [8, 10, 12] reported the change in early satiety score from baseline to week 4. One trial [12] reported the early satiety score at week 4. A total of 924 patients were included in the analysis (*P* = 0.20, *I*^2^ = 32.4%). The pooled results showed that compared with sham acupuncture, acupuncture could significantly reduce the early satiety score at week 4 (SMD -0.51, 95% CI -0.64 to -0.37) (Figure 9).

### 3.4. Sensitivity Analysis

**Figure 10:**
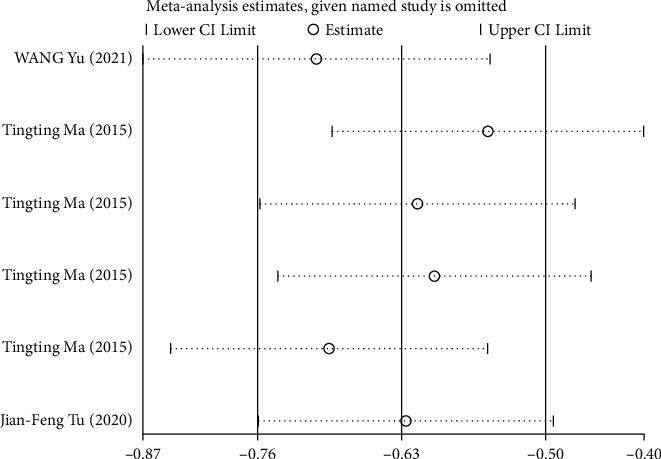
Sensitivity analysis of postprandial fullness.

We conducted sensitivity analysis on postprandial fullness by excluding literature one by one, and the analysis results showed that when a certain study was removed, the combined effect size of the remaining studies was still within the confidence interval, indicating that the sensitivity of postprandial fullness index was small and the analysis results were stable (Figure 10).

## 4. Discussion

Our study is the first systematic review of the effects of acupuncture in the treatment of PDS. The results showed that acupuncture could alleviate clinical symptoms in patients with PDS. Compared with the sham operation group, acupuncture could improve OTE at week 4 (SMD 4.74, 95% CI 02.88 to 7.83). At the same time, compared with the sham operation group, the NDI score was significantly improved, SID score was markedly decreased, and postprandial fullness and early satiety were also remarkably relieved in the acupuncture group. These data supported that acupuncture treatment could be a therapeutic strategy ameliorating the clinical symptoms of PDS patients. Based on the Rome IV consensus, the most typical symptoms of PDS are postprandial fullness (PPF) and/or early satiety (ES), [13] which is often triggered or aggravated after eating [14]. A systematic review [15] demonstrated that acupuncture could relieve dyspepsia symptoms and improve quality of life in patients with FD. Acupuncture therapy showed great benefits in regulating gastric motility, gastric accommodation, mental status, gastrointestinal hormones, and central and autonomic functions. NDI scores [16], relevant symptoms (especially postprandial fullness and early satiation), and quality of life of FD patients were remarkably improved by acupuncture compared with conventional medication (prokinetic agents) [17]. Acupuncture exerted potent effect in alleviating FD symptoms compared to domperidone or itopride [18]. In the acupuncture group, the Nepean dyspepsia index score and total effective rate were significantly improved, and the recurrence rate was greatly reduced after 3–6 months of follow-up than the Western medication (WM) group. Acupuncture in combination with conventional WM has the potential to improve FD treatment [19]. Another meta-analysis showed that combination of acupuncture and clebopride might be the most effective treatment for FD symptoms [18]. According to a recent meta-analysis [20], through genometic studies, acupuncture has been found to modulate gene expression and may be associated with the improvement of PDS symptoms. We can also see that with the introduction of metabolomics and genomics into the study of acupuncture treatment of diseases, although there is not much research in these two areas, acupuncture research has been brought to an increasingly micro level. At the same time, the central nervous and endocrine systems connect with each other in the role of acupuncture in the treatment of PDS also gradually revealed, the correlation of the few ways in the future we should clear what started out as a genetic regulation step by step already achieved in the treatment of eye, or several ways to play their own role, promote the improvement of gastric function. Acupuncture showed better effects in improving symptoms compared with pharmacotherapy (prokinetic agents (itopride, domperidone), 5-HT4 agonists (mosapride), antispasmodic calcium antagonist (pinaverium bromide), antidiarmodials (loperamide hydrochloride), Bifidobacterium, and antidepressants (flupentixol, melitracen). Some meta-analyses [21–23] suggested that acupuncture might be an effective and safe treatment for FD, but the quality of the included studies was low. In a systematic analysis on clinical trials, H. pylori eradication improved symptoms in patients with functional dyspepsia in the follow-up period of more than 1 year but was not effective in the follow-up period of less than 1 year. Moreover, eight studies showed that H. pylori eradication therapy increased treatment-related side effects compared to noneradication therapy [24]. Studies with high-quality evidence suggested that H. pylori eradication therapy could improve or even cure FD symptoms, but the benefits are limited [25]. However, two studies [26, 27] showed that eradication of H. pylori was more effective against EPS than PDS. A pilot study [28] found that proton pump inhibitors (PPIs) could reduce postprandial symptoms in healthy participants, particularly postprandial fullness. Acupuncture can effectively improve the symptoms of PDS patients, which may be related to improving gastric hypersensitivity of PDS through the adrenaline pathway, inhibiting the hippocampal glutamate system, and regulating AMPK/TSC2/Rheb-mediated mTOR inhibition to change Ghrelin level. A Cochrane meta-analysis [29] of 18 randomized controlled trials reported that PPI was more effective than placebo on relieving dyspepsia symptoms in FD patients (risk ratio (RR) 0.88, 95% confidence interval (CI) 0.82 to 0.94). However, relevant studies [30, 31] have shown that PPIs had no significant effect on the treatment of PDS. Prokinetics may be effective for all subtypes of FD but only low quality of evidence supported this result [32]. A review of Cochrane's system suggested that there was no exact evidence proving the efficacy of prokinetics in the treatment of FD [33]. A systematic [34] review has demonstrated that acotiamide could effectively relieve symptoms of early satiety and postprandial fullness in patients with PDS. Tricyclic antidepressants may be an effective treatment for FD. However, there is a lack of high-quality clinical evidence and there are more adverse events compared to placebo [35, 36]. Limited evidence could support that psychotherapy is beneficial for patients with FD [37]. Herbal remedies might be effective and safe in treating FD and demonstrate comparable effect sizes for efficacy to conventional treatments, [38] Xiao Yao Pill and modified Ban Xia Xie Xin Decoction probably had great beneficial effect on ameliorating postprandial fullness [39].

Our meta-analysis showed that acupuncture was effective compared with the sham operation group in the treatment of PDS. Acupuncture can improve the overall treatment efficacy and the quality of life of patients and relieve postprandial distress syndrome and the symptoms of dyspepsia. Particularly, acupuncture showed obvious advantages in alleviating the symptoms of early satiety and postprandial fullness. Our results supported that acupuncture was an effective therapeutic strategy for postprandial distress syndrome.

To our knowledge, this is the first meta-analysis on acupuncture treatment for PDS. The studies included in this analysis were of high quality, and the heterogeneity was not significant between groups. No obvious publication bias was found. A limitation of this meta-analysis was that the study on acupuncture treatment of PDS was relatively new. Although the relevant database was systematically searched, the sample sizes were relatively small, and 2 studies [9, 12] were published in Chinese. Unfortunately, our study did not evaluate the safety of acupuncture in the treatment of PDS.

This meta-analysis demonstrated that acupuncture was indeed an effective method to treat PDS compared with the sham acupuncture group. Acupuncture is a traditional approach to treat diseases and widely used in East Asian countries such as China, Japan, and South Korea. However, acupuncture is less commonly used in other regions and countries. More high-quality, multicenter, multiethnic studies were required to provide potent clinical evidence validating the efficacy of acupuncture in the treatment of PDS.

## 5. Conclusions

This study highlighted that the acupuncture could significantly improve the overall therapeutic effect of PDS patients, alleviate the symptoms of postprandial fullness and early satiety, and improve the quality of life of patients. Our results supported that acupuncture was an effective therapeutic strategy for postprandial distress syndrome.

## Figures and Tables

**Table 1:** Basic characteristics of included trials.

Authors	Published years	Study design	Number of patients		Age (year)		Disease duration		Interventions	
			Observation group	Control group	Observation group	Control group	Observation group	Control group	Observation group (acupuncture)	Control group (sham acupuncture)
Tu et al. [9]	2019	RCT	26	25	43 ± 17	41 ± 16	42.7 ± 54.1	59.9 ± 65.9	Acupuncture, acupoint selection GV20 (Baihui), CV12 (Zhongwan), CV6 (Qihai), CV17 (Danzhong), ST25 (Tianshu), PC6 (Neiguan), ST36 (Zusanli), and SP4 (Gongsun).	Sham acupuncture, nonacupoints
Stanghellini et al. [12]	2021	RCT	138	140	41.6 ± 13.1	41.2 ± 13.1	57.3 ± 63.7	61.6 ± 64.4	Acupuncture, acupoint selection GV20 (Baihui), CV12 (Zhongwan), CV6 (Qihai), CV17 (Danzhong), ST25 (Tianshu), PC6 (Neiguan), ST36 (Zusanli), and SP4 (Gongsun).	Sham acupuncture, nonacupoints
Kim et al. [8]	2015	RCT	79	71	38.8 ± 13.8	36.0 ± 13.0	75.7 ± 74.5	67.3 ± 80.3	Acupuncture, acupoint selection ST42 (Chongyang), ST40 (Fonglong), ST36 (Zusanli), and ST34 (Liangqiu)	Sham acupuncture, nonacupoints
Kim et al. [8]	2015	RCT	82	71	34.9 ± 13.9	36.0 ± 13.0	59.3 ± 52.2	67.3 ± 80.3	Acupuncture, acupoint selection ST38 (Tiaokou), ST35 (Dubi), ST33 (Yinshi), and ST32 (Futu)	Sham acupuncture, nonacupoints
Kim et al. [8]	2015	RCT	74	71	35.5 ± 11.6	36.0 ± 13.0	63.6 ± 60.1	67.3 ± 80.3	Acupuncture, acupoint selectionBL21 (Weishu) and CV12 (Zhongwan)	Sham acupuncture, nonacupoints
Kim et al. [8]	2015	RCT	85	71	38.5 ± 13.5	36.0 ± 13.0	70.4 ± 60.7	67.3 ± 80.3	Acupuncture, acupoint selection GB40 (Qiuxu), GB37 (Guangming), GB36 (Waiqiu), and GB34 (Yanglingquan)	Sham acupuncture, nonacupoints
Yang et al. [10]	2020	RCT	21	21	44.8 ± 13.3	46.0 ± 13.2	36 ± 26.67	54 ± 71.11	Acupuncture, acupoint selection GV20 (Baihui), CV12 (Zhongwan), CV6 (Qihai), CV17 (Danzhong), ST25 (Tianshu), PC6 (Neiguan), ST36 (Zusanli), and SP4 (Gongsun).	Sham acupuncture, nonacupoints
Wang et al. [11]	2020	RCT	138	140	41.6 ± 13.1	41.2 ± 13.1	57.3 ± 63.7	61.6 ± 64.4	Acupuncture, acupoint selection GV20 (Baihui), CV12 (Zhongwan), CV6 (Qihai), CV17 (Danzhong), ST25 (Tianshu), PC6 (Neiguan), ST36 (Zusanli), and SP4 (Gongsun).	Sham acupuncture, nonacupoints

RCT: randomized controlled trial; NDI: Nepean dyspepsia index; SID: symptom index of dyspepsia.

## Data Availability

The data and materials in the current study are available from the corresponding author on reasonable request.
